# Efficacy and feasibility of deep brain stimulation for patients with depression: A protocol for systematic review and meta-analysis

**DOI:** 10.1097/MD.0000000000026044

**Published:** 2021-05-21

**Authors:** Hongli Zhang, Na Wang, Liping Yu, Min Zhao

**Affiliations:** a Institute of Psychology; b Institute of Management, Weifang Medical University, Shandong, China.

**Keywords:** deep brain stimulation, depression, meta-analysis, protocol, review

## Abstract

**Background:**

Previous meta-analyses have examined the clinical efficacy and acceptability of deep brain stimulation (DBS) compared with sham therapy or paired active therapy. However, the absence of head-to-head clinical trials with some treatment comparisons creates uncertainty for decision makers. Thus, to provide new evidence-based medical evidence for clinical treatment, we undertook a meta-analysis to assess the efficacy and safety of DBS in patients with depression based on high-quality randomized controlled studies.

**Methods:**

The protocol was written following the Preferred Reporting Items for Systematic Reviews and Meta-Analyses Protocols (PRISMA-P) statement guidelines. PubMed/Medline and EMBASE will be searched before May 2021 for all studies, using various combinations of the following free text and key terms: deep brain stimulation; depression; random. No language restrictions will be applied. The method of data extraction will follow the approach outlined by the Cochrane Handbook for Systematic Reviews of Interventions. Review Manager software 5.3 is used for the meta-analysis. The quality of randomized trials will be assessed by Cochrane risk of bias tool for randomized controlled trials.

**Results:**

The results of our review will be reported strictly following the PRISMA criteria and the review will add to the existing literature by showing compelling evidence and improved guidance in clinic settings.

**OSF registration number:**

10.17605/OSF.IO/Q5B3S.

## 1 Introduction

Depression affects >17 million people in the United States, and these disorders are a significant contributor to poor quality of life. Depression is a severe psychiatric disorder with recurring episodes, with each episode increasing the risk of subsequent episodes by about 20 percent each year.^[1]^ Treatment for these conditions includes drug combination therapy, alternative psychotherapy, physical therapy, and even ablative psychosurgery. Even so, 10% to 20% of patients remain depressed or at risk of relapse.^[2–4]^

Presently, interest in the psychiatric treatment of neurological disorders is shifting from ablative psychosurgical procedures, which aim to destroy brain tissue, to deep brain stimulation (DBS), which aims to stimulate brain regions through implanted electrodes.^[5,6]^ An optimal approach has yet to be established, as the neuropathophysiology of depression remains weakly defined, and the mechanism of DBS seems to be dependent on the stimulation site. The optimal target, stimulus parameters and stimulus package have yet to be determined. DBS has shown preliminary evidence of an antidepressant effect in open-label studies and is still considered investigational in treatment guidelines.^[7,8]^

Previous meta-analyses have examined the clinical efficacy and acceptability of DBS compared with sham therapy or paired active therapy.^[9,10]^ These methods provide only limited insight into the overall treatment approach, since treatment effects are estimated based on only a subset of relevant treatment comparisons and are provided only for a subset of relevant treatment comparisons. In addition, the absence of head-to-head clinical trials with some treatment comparisons creates uncertainty for decision makers. Thus, to provide new evidence-based medical evidence for clinical treatment, we undertook a meta-analysis to assess the efficacy and safety of DBS in patients with depression based on high-quality randomized controlled studies.

## 2 Materials and methods

### 2.1 Searching strategy

The protocol was written following the Preferred Reporting Items for Systematic Reviews and Meta-Analyses Protocols (PRISMA-P) statement guidelines. PubMed/Medline and EMBASE will be searched before May 2021 for all studies, using various combinations of the following free text and key terms: deep brain stimulation; depression; random. No language restrictions will be applied. We will also search citations of relevant primary and review. Authors of abstract in the meeting will be further searched in PubMed for potential full articles. To minimize the risk of publication bias, we will conduct a comprehensive search that included strategies to find published and unpublished studies. The prospective registration has been approved by the Open Science Framework. Ethical approval is not necessary because the present meta-analysis will be performed based on previously published studies.

### 2.2 Eligibility criteria

Study included in this review has to meet all of the following inclusion criteria in the PICOS order:

1. population: patients with depression;
2. intervention group (group 1): DBS group;
3. comparison group (group 2): control group with no DBS;
4. outcome measures: the primary outcome measure was clinical response, defined as a ≥50% reduction in symptom scores at the primary study endpoint. Remission rates were the secondary outcome measure based on the definition provided by each study;
5. study design: randomized controlled trial.

Biomechanical studies, in vitro studies, review articles, techniques, case reports, letters to the editor, and editorials are excluded.

### 2.3 Study selection

The first author will conduct a preliminary screening based on the title to eliminate any research not related to the topic. A log of excluded studies is kept with the rationale for exclusion. Subsequently, all remaining abstracts will be reviewed by the primary author, and the selection criteria are applied. Studies identified for full text review will be evaluated by 2 authors for inclusion in the study. Disagreements will be resolved through a discussion with a third review author. Journal titles and authors’ names will be not glossed over in the research selection process. A manual search of the bibliographies of included studies is performed to ensure that the overall search was comprehensive and complete.

### 2.4 Data extraction

The method of data extraction will follow the approach outlined by the Cochrane Handbook for Systematic Reviews of Interventions. Two independent authors will extract the following descriptive raw information from the selected studies: study characteristics such as the first author, publication year, study design, follow-up period; patient demographic details such as patients’ number, average age, and sex ratio. The corresponding author will be contacted and asked to provide the data if the SD is not reported. In the case of no response, the SD will be calculated from the available data according to the previously validated formula: (higher range value – lower range value)/4 or interquartile range/1.35. The highest SD will be used if the SD cannot be calculated using this approach. If necessary, we will abandon the extraction of incomplete data.

### 2.5 Statistical analysis

Review Manager software 5.3 is used for the meta-analysis. Extracted data are entered into Review Manager by the first independent author and checked by the second independent author. Risk ratio with a 95% confidence interval or standardized mean difference with 95% CI are assessed for dichotomous outcomes or continuous outcomes, respectively. The heterogeneity is assessed by using the *Q* test and *I*^2^ statistic. An *I*^2^ value of <25% is chosen to represent low heterogeneity and an *I*^2^ value of >75% to indicate high heterogeneity. All outcomes are pooled on random-effect model. A *P* value of <0.05 is considered to be statistically significant.

### 2.6 Quality evaluation

The quality of randomized trials will be assessed by Cochrane risk of bias tool for randomized controlled trials. Each article will be reviewed by one reviewer and verified by a second and disagreements will be resolved by discussion with a third reviewer. A meta-analysis will be conducted when ≥3 trials reported an outcome of interest. We also will perform the sensitivity analysis to evaluate whether the differences of study design had an impact on the overall estimate and data. Review Manager software 5.3 will be conducted for statistical investigation and a funnel plot analysis will be drawn to assess the publication bias if there are more than 10 studies included.

## 3 Discussion

Previous meta-analyses have examined the clinical efficacy and acceptability of DBS compared with sham therapy or paired active therapy.^[9,10]^ These methods provide only limited insight into the overall treatment approach, since treatment effects are estimated based on only a subset of relevant treatment comparisons and are provided only for a subset of relevant treatment comparisons. In addition, the absence of head-to-head clinical trials with some treatment comparisons creates uncertainty for decision makers. Thus, to provide new evidence-based medical evidence for clinical treatment, we undertook a meta-analysis to assess the efficacy and safety of DBS in patients with depression based on high-quality randomized controlled studies. For this study, our review process will be very rigorous. And this article is a protocol of the systematic review and meta-analysis, which presents the detailed description of review implement. The results of our review will be reported strictly following the PRISMA criteria and the review will add to the existing literature by showing compelling evidence and improved guidance in clinic settings.

## Author contributions

**Conceptualization:** Liping Yu.

**Data curation:** Hongli Zhang, Na Wang.

**Formal analysis:** Hongli Zhang, Na Wang.

**Funding acquisition:** Min Zhao.

**Investigation:** Hongli Zhang, Na Wang.

**Methodology:** Liping Yu, Na Wang.

**Project administration:** Min Zhao.

**Resources:** Liping Yu, Min Zhao.

**Software:** Liping Yu, Na Wang.

**Supervision:** Min Zhao.

**Validation:** Hongli Zhang, Liping Yu.

**Visualization:** Liping Yu.

**Writing – original draft:** Hongli Zhang.

**Writing – review & editing:** Min Zhao.
